# Desvendando a Perícia na Substituição da Válvula Aórtica por Cateter no Brasil: Lições dos Dados Nacionais

**DOI:** 10.36660/abc.20240302

**Published:** 2024-07-23

**Authors:** Sofia Cabral

**Affiliations:** 1 Centro Hospitalar Universitário de Santo António Porto Portugal Centro Hospitalar Universitário de Santo António, Porto - Portugal

**Keywords:** Curva de Aprendizado, Substituição da Valva Aórtica Transcateter, Brasil

*"Pois as coisas que temos que aprender antes de podermos fazê-las, aprendemos fazendo-as"*

Aristóteles

Os resultados dos procedimentos em cardiologia, como em outras áreas da medicina, são influenciados por vários fatores, incluindo características do paciente, complexidade do procedimento, proficiência do operador, diferenciação tecnológica disponível e recursos de saúde. Centros de maior volume relatam melhores resultados para muitos procedimentos, embora a extensão desse benefício varie dependendo da complexidade do procedimento. Quanto mais complexo for o procedimento, maior será a relação entre experiência e resultados. Além disso, cada procedimento tem sua curva de aprendizado (CA), tanto ao nível individual quanto em grupo, o que introduz densidade a esta avaliação. Do ponto de vista acadêmico, uma CA define a relação entre desempenho (aprendizagem) e prática (esforço). Na formação em saúde, este processo de aprendizagem segue tipicamente um padrão não linear inicialmente marcado por um declive acentuado à medida que os princípios básicos do procedimento são adquiridos, seguido por um ponto de inflexão onde a taxa de aprendizagem desacelera. Eventualmente, atinge uma expressão assintótica, representando a fase de platô de alta proficiência.^1^ Compreender como se comporta a CA de um procedimento é importante porque fornece informações valiosas para as sociedades científicas e as autoridades reguladoras de saúde na definição dos requisitos de competência para os profissionais e auxiliando os tomadores de decisão na alocação eficaz de recursos. Mais importante ainda, desempenha um papel crucial na manutenção de elevados padrões de cuidados.

No campo das intervenções cardiovasculares, o fenômeno CA tem sido amplamente verificado, desde a cirurgia cardíaca^2^ à intervenção coronária percutânea,^3^ e, mais recentemente, a substituição transcateter da válvula aórtica (TAVI).^4-7^ Apesar dos resultados conflitantes de diferentes séries, os investigadores reconheceram um padrão consistente na CA da TAVI. Este padrão apresenta uma fase inicial em que a eficiência do procedimento e os resultados de segurança aumentam acentuadamente com o aumento da experiência, seguida por fases de estabilização e eventuais fases de consolidação.^6^

A maior parte dos dados provém de sistemas de saúde ocidentais de países de elevado rendimento, com informações limitadas disponíveis sobre o seu padrão noutras regiões geográficas e em países com economias mais débeis.^8^

Em seu estudo, Bernardi et al.^9^ tiveram como objetivo avaliar se a CA conhecida para resultados intra-hospitalares na TAVI é replicada no Brasil, servindo como proxy de uma nação sul-americana de renda baixa a média.^9^ Os autores utilizaram dados do registro nacional de TAVI no Brasil, abrangendo registros de 3.194 pacientes de 2008 a 2023. Empregando metodologia semelhante a Russo et al.,^7^ eles descobriram que após o ajuste para o Euroscore e o modelo de prótese (modelos mais recentes versus modelos de primeira geração), a mortalidade hospitalar exibiu um declínio inicial após o número de sequência de casos (CS #) 40, se estabilizou em CS #118 e atingiu seu ponto final no CS #303, além do qual nenhum outro benefício marginal tangível foi perceptível. Este comportamento dos dados segue uma CA típica.

Além disso, os investigadores realizaram uma subanálise paralela comparando centros com base na adoção precoce ou tardia do procedimento, usando 2014 como marco temporal de referência, **que coincidiu com a introdução de próteses de nova geração no mercado brasileiro**. Surpreendentemente, mas não totalmente inesperado, foi observada uma CA distinta para a coorte de adotantes iniciais, mas não para a contraparte de adotantes tardios. Esses achados questionam o fenômeno da CA na prática atual do TAVI e estão de acordo com os relatados por Russo et al.^7^ O menor impacto da experiência nos resultados iniciais em programas de TAVI mais recentes pode ser atribuído a avanços tecnológicos, maturação da técnica, programas sólidos estruturados e conhecimento compartilhado acumulado. Na verdade, poucas áreas da medicina cardiovascular testemunharam uma supervisão tão intensiva como os procedimentos TAVI, um aspecto notável que não deve ser subestimado como contribuinte. Os autores reconheceram plenamente todos estes aspectos, demonstrando uma perspicácia apurada.

Outro ponto crucial a considerar é que, em todo o grupo, a mortalidade hospitalar foi impactada pela experiência. A maior experiência, definida como mais de 120 casos acumulados, levou inequivocamente a melhores resultados. No entanto, há uma ressalva neste achado: nos adotantes tardios, o efeito da elevada experiência na mortalidade hospitalar foi imperceptível. As diferenças encontradas na subanálise das duas coortes, onde os adotantes tardios não conseguiram reproduzir a tendência global, estão provavelmente enraizadas no seu menor volume médio de casos acumulados (56 vs. 222; intervalo interquartil de 34,5 a 118), bem abaixo do limiar de elevada experiência (120) onde foi observado um claro benefício em termos de mortalidade. Isto sugere que este grupo provavelmente experimentou uma CA amortecida em vez de sua completa ausência, relacionada ao efeito de dispersão do aumento da disponibilidade do TAVI no volume de procedimentos de cada centro ao longo do tempo. A implicação prática é que deve haver um equilíbrio delicado entre garantir o acesso oportuno da população ao procedimento e garantir a sua eficácia. Felizmente, durante a sua experiência inicial (primeiro quartil), as taxas de mortalidade hospitalar dos adoptantes tardios situaram-se entre as encontradas no segundo e terceiro quartis de experiência acumulada entre os adoptantes precoces. Isto aponta para uma verdadeira modulação da CA na prática contemporânea da TAVI.

A investigação conduzida por Bernardi et al.^9^ é valiosa porque retrata a implementação da TAVI no Brasil e fornece um caminho para explorar os desafios e fatores específicos em jogo em um cenário local. Tal como noutros contextos económicos e sociais, é fundamental reconhecer que a influência da experiência nos resultados dos programas TAVI transcende a mera proficiência técnica. Estende-se a aspectos mais amplos da saúde, combinando atendimento ao paciente, planejamento de procedimentos e gerenciamento periprocedimento - determinantes que este estudo não foi projetado para capturar. Não obstante, muitos insights valiosos emergem deste estudo, extraído do Registro Brasileiro de TAVI, que pode ajudar a moldar a prestação de TAVI no Brasil.
