# Supplementary material for: Desvendando a Perícia na Substituição da Válvula Aórtica por Cateter no Brasil: Lições dos Dados Nacionais
Source: Arq Bras Cardiol. 2024 Jul 23;121(7):e20240302. [Article in Portuguese] doi: 10.36660/abc.20240302 (PMC11341207; doi:10.36660/abc.20240302)
Supplement: Translation

# Translation: Unlocking Transcatheter Aortic Valve Replacement Expertise in Brazil: Lessons from National Data

**DOI:** 10.36660/abc.20240302i

**Authors:** Sofia Cabral

**Affiliations:** 1 Porto Portugal Centro Hospitalar Universitário de Santo António, Porto - Portugal

**Keywords:** Learning Curve, Transcatheter Aortic Valve Replacement, Brazil

*"For the things we have to learn before we can do them, we learn by doing them"*

*Aristotle*

Procedural outcomes in cardiology, as in other fields in Medicine, are influenced by numerous factors, including patient characteristics, procedural intricacy, operator proficiency, available technological differentiation, and healthcare resources. Higher volume centers report better results for many procedures, though the extent of that benefit varies depending on the procedure's complexity. The more complex the procedure the higher the relationship between experience and outcomes. Additionally, each procedure has its learning curve (LC), both at individual and group levels, which adds density to the assessment. From an academic standpoint, an LC defines the relationship between performance (learning) and practice (effort). In health training, this learning process typically follows a non-linear pattern initially marked by a steep slope as the basics of the procedure are acquired, followed by an inflection point where the learning rate decelerates. Eventually, it reaches an asymptotic expression, representing the plateau phase of high proficiency.^1^ Understanding how a procedure's LC behaves is important because it provides valuable insights for scientific societies and healthcare regulatory authorities in defining the competency requirements for practitioners and assisting decision-makers in effective resource allocation. Most importantly, it plays a critical role in maintaining high standards of care.

In the field of cardiovascular interventions, the LC phenomenon has extensively been verified, from cardiac surgery^2^ to coronary percutaneous intervention,^3^ and more recently, transcatheter aortic valve replacement (TAVR).^4-7^ Despite conflicting results from different series, researchers have consistently recognized a pattern in the LC of TAVR. This pattern depicts an early phase where procedure efficiency and safety outcomes steeply increase with increasing experience, followed by flattening out and eventual consolidation phases.^6^

Most of the data comes from Western, high-income healthcare systems, with limited information available on its pattern in other geographic regions and economically constrained countries.^8^

In their study, Bernardi et al.^9^ aimed to assess whether the established LC for in-hospital outcomes in TAVR is replicated in Brazil, serving as a proxy of a South American low-to-middle-income nation.^9^ The authors utilized data from the Brazilian nationwide TAVR registry, covering records from 3194 patients spanning from 2008 to 2023. Employing a methodology similar to Russo MJ et al.,^7^ they discovered that after adjusting for Euroscore and prosthesis model (newer vs. first-generation models), in-hospital mortality exhibited an initial decline after case-sequence number (CS #) 40, plateaued at CS #118, and reached its termination point at CS #303, beyond which no further tangible marginal benefit was noticeable. This data expression follows a typical LC.

Additionally, the investigators conducted a parallel sub-analysis comparing centers based on early or late adoption of the procedure, using 2014 as the reference time mark, **which coincided with the introduction of new-generation prostheses in the Brazilian market**. Surprisingly but not entirely unexpected, a distinct LC was observed for the early-adopters cohort, but not for the late-adopters counterpart. These findings question the LC phenomenon in current TAVR practice and are in keeping with those reported by Russo et al.^7^ The lesser impact of experience on early outcomes in more recent TAVR programs can be attributed to technological breakthroughs, maturation of the technique, sound structured programs, and accumulated shared knowledge. Indeed, few areas in cardiovascular medicine have witnessed such intensive proctorship as TAVR procedures, a notable aspect that should not be underestimated as a contributor. The authors fully acknowledged all these aspects, demonstrating keen awareness.

Another crucial point to consider is that, within the entire group, in-hospital mortality was impacted by experience. Higher experience, defined as more than 120 accumulated cases, unequivocally led to better outcomes. However, there's a caveat to this finding: in late adopters, the effect of high experience on in-hospital mortality was unnoticeable. The differences found in the two-cohort subanalysis, where late adopters failed to reproduce the global trend, are probably rooted in their lower median accumulated case volume (56 vs 222; interquartile range from 34,5 to 118), well below the threshold of high experience (120) where a clear mortality benefit was observed. This suggests that this group most likely experienced a dampened LC rather than its complete absence, related to the scattering effect of the increased TAVR availability on each center's procedural volume over time. The practical implication is that there must be a delicate balance between ensuring the population's timely access to the procedure and guaranteeing its effectiveness. Reassuringly, during their early experience (first quartile), late adopters’ in-hospital mortality rates fell in between those found in the second and third quartiles of accumulated experience among early adopters. This argues for a true reshaping of the LC in contemporary TAVR practice.

The investigation conducted by Bernardo et al.^9^ is valuable as it portrays the implementation of TAVR in Brazil and provides an avenue to explore the specific challenges and factors at play in a local setting. As in other economic and social contexts, it is paramount to recognize that the influence of experience on the outcomes of TAVR programs transcends mere technical proficiency. It extends to broader aspects of healthcare, blending patient care, procedural planning, and peri-procedural management -determinants that this study wasn't designed to capture. Notwithstanding, plenty of valuable insights emerge from this study, drawn from the Brazilian TAVR Registry, which can assist in shaping TAVR delivery in Brazil.
